# Spexin disrupts migrating myoelectric complex in the rat small intestine: The role of galanin-2 and muscarinic receptors

**DOI:** 10.1007/s00424-026-03180-1

**Published:** 2026-05-19

**Authors:** Özge Darakcı Saltık, Hakan Balcı, Rümeysa Abdullahoğlu, Ayhan Bozkurt

**Affiliations:** 1 Faculty of Medicine, Department of Physiology, Bilecik Şeyh Edebali University, Bilecik, Turkey; 2 Faculty of Medicine, Department of Medical Pharmacology, Bilecik Şeyh Edebali University, Bilecik, Turkey; 3 Faculty of Medicine, Department of Physiology, Ondokuz Mayıs University, Samsun, Turkey

**Keywords:** Galanin-2 receptor, Intestinal motility, Migrating myoelectric complex, Muscarinic receptor, Spexin, Rat

## Abstract

Spexin (SPX) is an endogenous peptide expressed throughout the gastrointestinal tract. Although its impact on postprandial intestinal motility has been examined, its effect during fasting remains unclear. This study aimed to investigate the direct effects of SPX on jejunal and ileal segments *in vitro*, its effects on fasting small intestinal motility *in vivo*, and to determine the roles of galanin-2, muscarinic, and 5-hydroxytryptamine-3 (5-HT₃) receptors in these actions. The contractile responses of rat jejunal and ileal segments to SPX (10⁻⁹–10⁻⁶ M), with or without pretreatment with galanin-2, muscarinic, or 5-HT₃ receptor antagonists (M871, atropine, and ondansetron, respectively) were evaluated in organ baths. For *in vivo* experiments, bipolar electrodes were implanted at two jejunal sites to record migrating myoelectric complexes (MMC), and a catheter was inserted into the left jugular vein for drug administration. SPX (40–640 µg/kg/h) was infused for 1 hour following basal MMC recording. Antagonists were administered 5 minutes prior to SPX (320 µg/kg/h) infusion. SPX induced concentration-dependent contractions in both intestinal segments, significantly inhibited by M871 but unaffected by atropine or ondansetron. At 160 μg/kg/h, SPX altered the MMC pattern; at 320 μg/kg/h, it disrupted MMC and induced irregular spiking activity, which was blocked by M871 and atropine, but not by ondansetron. These data indicate that SPX modulates fasted motility pattern, involving GALR2-dependent mechanisms and an indirect contribution of muscarinic pathways. These findings may support the development of therapies for gastrointestinal motility disorders.

**Graphical abstract:**

*In vitro*, SPX induced concentration-dependent contractions, which were inhibited by M871 but not affected by atropine or ondansetron. *In vivo*, SPX disrupted the fasting intestinal myoelectric pattern, an effect that was blocked by M871 and atropine but not by ondansetron.

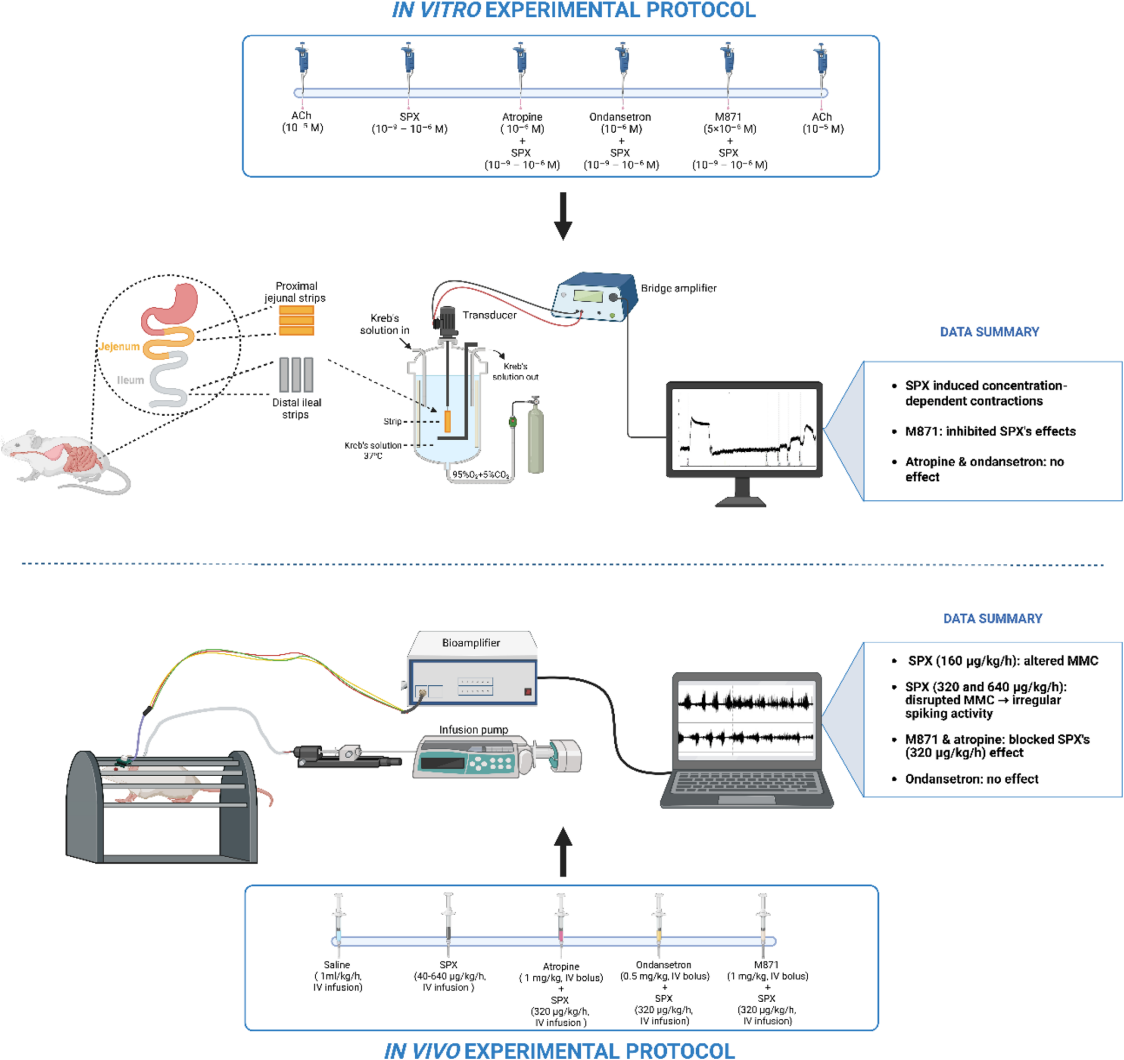

## Introduction

Spexin (SPX), a 14-amino-acid neuropeptide, was discovered in 2007 through bioinformatic analyses [43]. It is widely expressed in the central nervous system and peripheral tissues, including the hypothalamus, pituitary gland, gastrointestinal tract, endocrine glands, and reproductive organs [37, 41]. Due to its broad distribution in various tissues—especially in neurons and endocrine cells—spexin is proposed to act as a neurotransmitter, neuromodulator, or endocrine signaling molecule. Exogenously administered SPX suppresses feeding by acting on hypothalamic centers [21, 45, 51, 55], and affects lipid and glucose metabolism [14]. SPX has been shown to reduce various types of pain in animal models, including somatic, inflammatory, visceral, and neuropathic pain [30, 33, 35, 47]. Additionally, it modulates the release of endocrine signaling molecules gonadotropin-releasing hormone, luteinizing hormone, follicle-stimulating hormone, ghrelin, leptin, corticosterone, triiodothyronine, and glucagon [12, 29, 50, 52]. Despite its demonstrated effects in a wide range of physiological processes, the specifically attributed receptor for spexin remains conclusively unidentified. As a ligand for the galanin receptor subfamily, SPX exerts its effects by activating galanin-2 receptor (GALR2) and galanin-3 receptor (GALR3) while showing no affinity for galanin-1 receptor (GALR1) [26].

Recent studies highlight the role of SPX in gastrointestinal motility, with SPX expression detected in the submucosal layers of the stomach and intestines. Experimental data demonstrate that SPX induces dose-dependent contractions in smooth muscle strips of the stomach and intestines [28, 34]. Moreover, its effect in the intestines is primarily mediated through activation of GALR2s [28]. Peripheral administration of SPX accelerates intestinal and colonic transit, reinforcing its role in motility regulation. Reduced serum SPX levels are linked to constipation and delayed intestinal transit, suggesting its promising potential as a biomarker for gastrointestinal dysmotility disorders [28]. However, while SPX’s role in postprandial motility is established, its potential to modulate fasting motility remains unexplored. Fasting motility in the small intestine follows a cyclic pattern known as the migrating motor/motility complex, which emerges during the interdigestive period to act as a ‘housekeeper’ by clearing accumulated debris, preventing bacterial overgrowth, and ensuring the proper propulsion of intestinal contents toward the colon [11, 22, 23]. The electrical activity underlying this cyclic pattern is referred to as ‘migrating myoelectric complex’ (MMC) [44]. Whether SPX influences MMC and thereby contributes to fasting small intestinal motility has not yet been elucidated.

Our study aims to evaluate the effect of SPX on isolated proximal jejunum and distal ileum smooth muscle segments, as well as the involvement of muscarinic, galaninergic and serotonergic receptors in this potential effect. This study further aims to investigate the impact of intravenously administered SPX on MMC and explores the receptor-specific contributions to SPX-induced fasting motility responses. To this end, both in vitro and in vivo experimental approaches were employed to comprehensively investigate the effects of SPX on small intestinal motility.

## Materials and methods

### Animals

This research was conducted at the Faculty of Medicine, Ondokuz Mayıs University, Samsun, Turkey. Ethical approval was obtained from the local Animal Studies Ethics Committee (HADYEK 2014/638), in compliance with national and international standards for animal research. The study adhered to the Guide for the Care and Use of Laboratory Animals (NIH Publication No. 865–23) and institutional protocols to minimize distress and ensure humane treatment. Male Sprague-Dawley rats (250–300 g) were housed under controlled temperature and humidity conditions with a 12–hour light/dark cycle. Food and water were provided *ad libitum*.

### In vitro studies

Fasted rats (*n* = 7) were anesthetized with intraperitoneal (IP) ketamine (100 mg/kg) and chlorpromazine (0.75 mg/kg). Following a midline abdominal incision, 2–3 cm intestinal segments were rapidly excised. Proximal jejunal segments were isolated 15 cm distal to the ligament of Treitz, and distal ileal segments were collected 15 cm proximal to the cecum. The isolated strips were suspended in 10 ml organ bath chambers containing oxygenated (95% O_2_ and 5% CO_2_) Krebs-Henseleit solution (119.8 mM NaCl, 4.6 mM KCl, 0.5 mM MgCl_2_, 1.5 mM NaH_2_PO_4_, 2.5 mM CaCl_2_, 0.7 mM Na_2_HPO_4_, and 10.0 mM D-glucose) at 37◦C. Baseline tension was adjusted to 1.5 g, and isometric contractions were recorded using a PowerLab data acquisition system (ADInstruments, Australia). Throughout the 1-hour equilibration period, tissue strips were washed every 15 min. At the end of this period, a 5-minute baseline recording was obtained to ensure stable contractile activity [17, 28].

### Experimental protocol for in vitro studies

In vitro experiments were conducted using sequential drug applications on the same tissue preparations, with adequate washout periods between treatments to minimize carryover effects. Accordingly, the experimental protocol consisted of the following procedures:

#### Set 1

Acetylcholine chloride (ACh, 10⁻⁵ M) was applied to intestinal strips to determine the maximum contraction response.

#### Set 2

Spexin was administered in cumulative concentrations ranging from 10⁻⁹ to 10⁻⁶ M.

#### Set 3

Atropine (10⁻⁶ M), a nonselective muscarinic receptor antagonist, was given 10 min before SPX administration.

#### Set 4

Ondansetron (10⁻⁶ M), a 5-HT_3_ receptor antagonist, was applied 10 min before SPX administration.

#### Set 5

M871 (5 × 10^− 8^ M), a GALR2 antagonist, was given 10 min before SPX administration.

#### Set 6

ACh (10⁻⁵ M) was applied again to verify tissue viability.

**Fig. 1:**
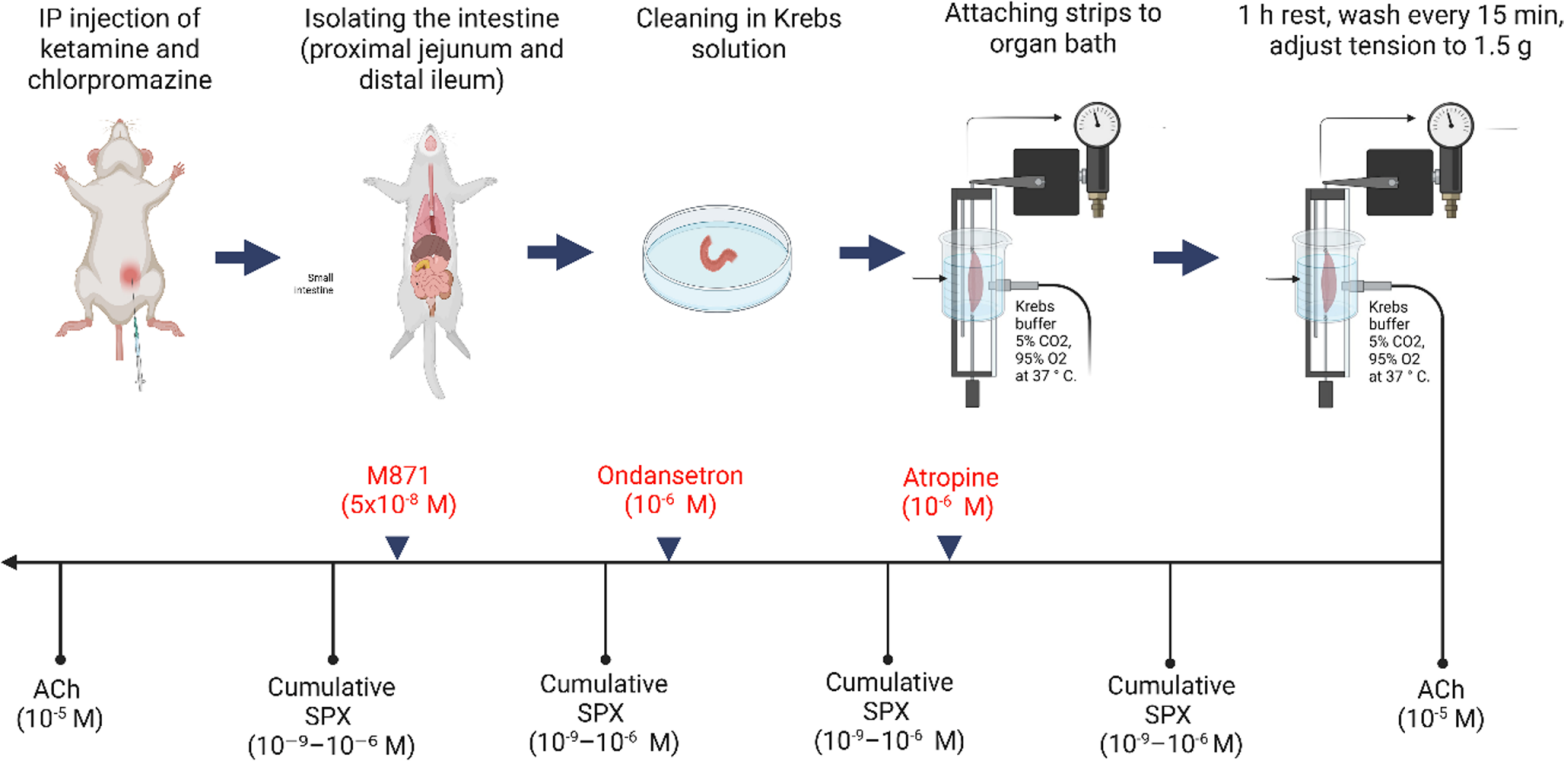
In vitro experimental protocols, showing preparation of intestinal strips and dose applications. The drug doses used in in vitro experiments were selected based on doses reported in the literature [2, 17, 28, 34]. Created with *BioRender.com*

Between each set, the strips were washed with fresh Krebs buffer and allowed to rest for 20 min. Figure 1 illustrates the preparation of the intestinal strips and the experimental procedure.

### In vivo electromyography studies

Twenty-four rats were anesthetized with ketamine (100 mg/kg, IP) and chlorpromazine (0.75 mg/kg, IP). A midline incision was made to place 80 μm Ni/Cr wire electrodes for electromyographic (EMG) recording at 15 cm (J1), 25 cm (J2) distal to the pylorus. A catheter was inserted into the right jugular vein for intravenous (IV) drug administration, followed by subcutaneous tunneling and securing of electrodes with dental acrylic to ensure stable positioning for long-term EMG recordings. Postoperative infection was prevented with cefazolin [100 mg/kg/day, intramuscular (IM)] administered for three days. Rats were acclimated to Bollman cages for 2 h (h) daily until the first day of EMG experiments (i.e., postoperative day 7). Before each EMG recording, the rats were fasted for 18 h to ensure a stable baseline fasted motility [6, 17, 18]. EMG recordings were conducted on conscious animals using a Bioamplifier (ML132, ADInstrument, Australia) and analyzed with PowerLab (ML870/P). Each experimental group included data from 7 to 9 observations. To achieve this, each animal was randomly assigned to multiple experimental groups (2–5 per animal), separated by a washout period of at least 3 days to minimize potential carryover effects. This approach was adopted in accordance with the principles of the 3Rs (Reduction, Replacement, and Refinement), aiming to reduce the number of animals used while minimizing the additional surgical burden associated with repeated electrode implantations.

### Experimental protocol for electromyography studies

**Fig. 2:**
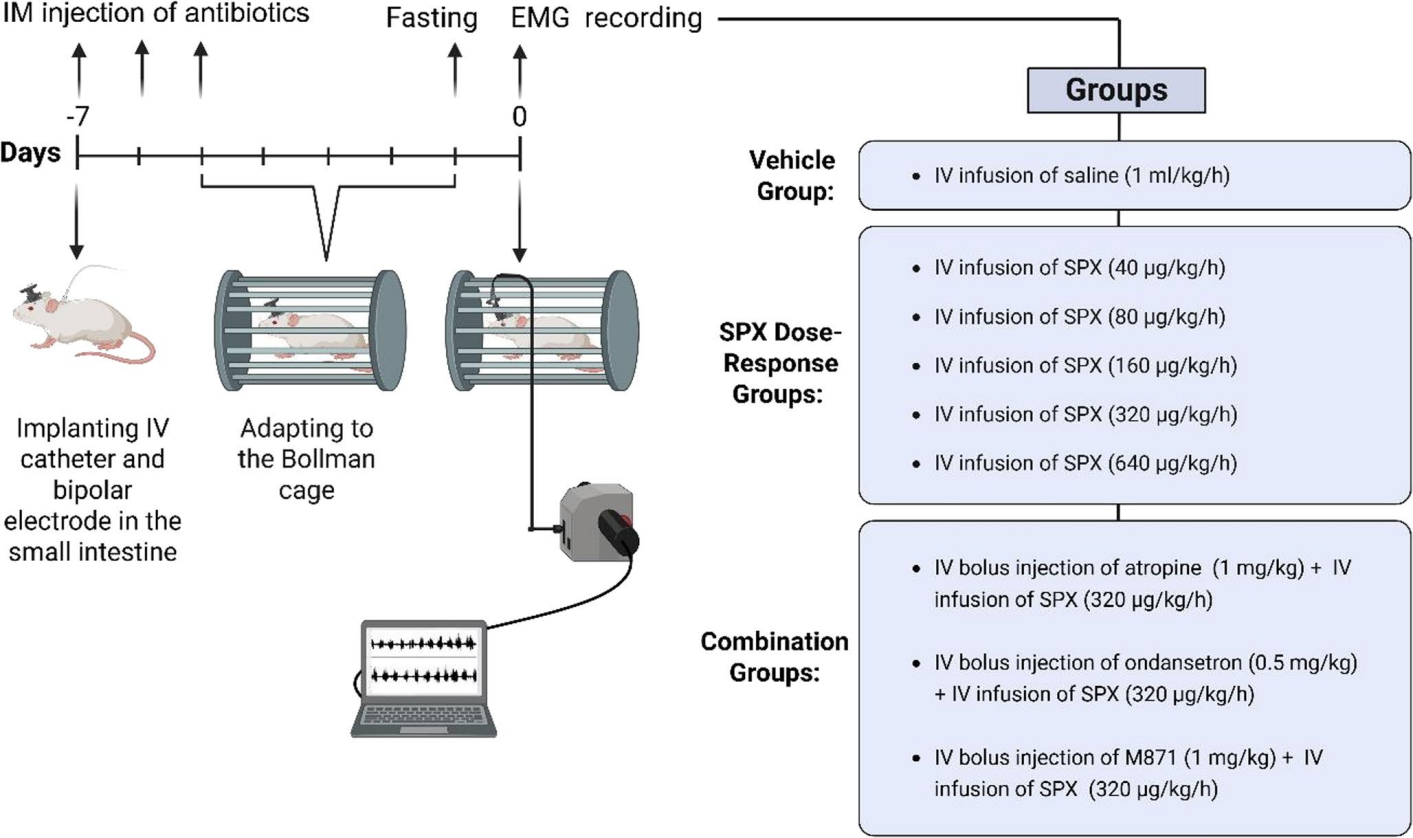
In vivo experimental protocols and groups (*n* = 7–9). The drug doses used in in vivo experiments were selected based on doses reported in the literature [28, 31, 49]. Created with *BioRender.com*

Experiments were started with a control recording of baseline myoelectric activity with three MMC cycles propagated across both sites (J1, J2) during a period of approximately 1 h. Following this period, SPX was administered as a continuous intravenous infusion (40–640 µg/kg/h for 1 h), initiated immediately after the completion of the fourth MMC cycle at the J1 site, with a total infusion volume of 1 mL. Antagonists were administered as intravenous bolus injections in a volume of 0.25 mL 5 min prior to SPX infusion. Experimental groups and drug administration protocols are illustrated in Fig. 2.

### Drugs

Spexin was purchased from PolyPeptide (Strasbourg, France); acetylcholine chloride, ondansetron, and atropine were obtained from Sigma-Aldrich (St. Louis, MO, USA); and M871 was sourced from Bachem (Bubendorf, Switzerland). All agents were dissolved in saline.

### Data analysis

In in vitro experiments, the dose of ACh that produced the maximum contractile effect in isolated small intestine segments was determined to be 10⁻⁵ M. ACh-induced responses were defined as 100%, and SPX responses in both intestinal segments were calculated as the percentage of the contractile response elicited by ACh.

**Fig. 3:**
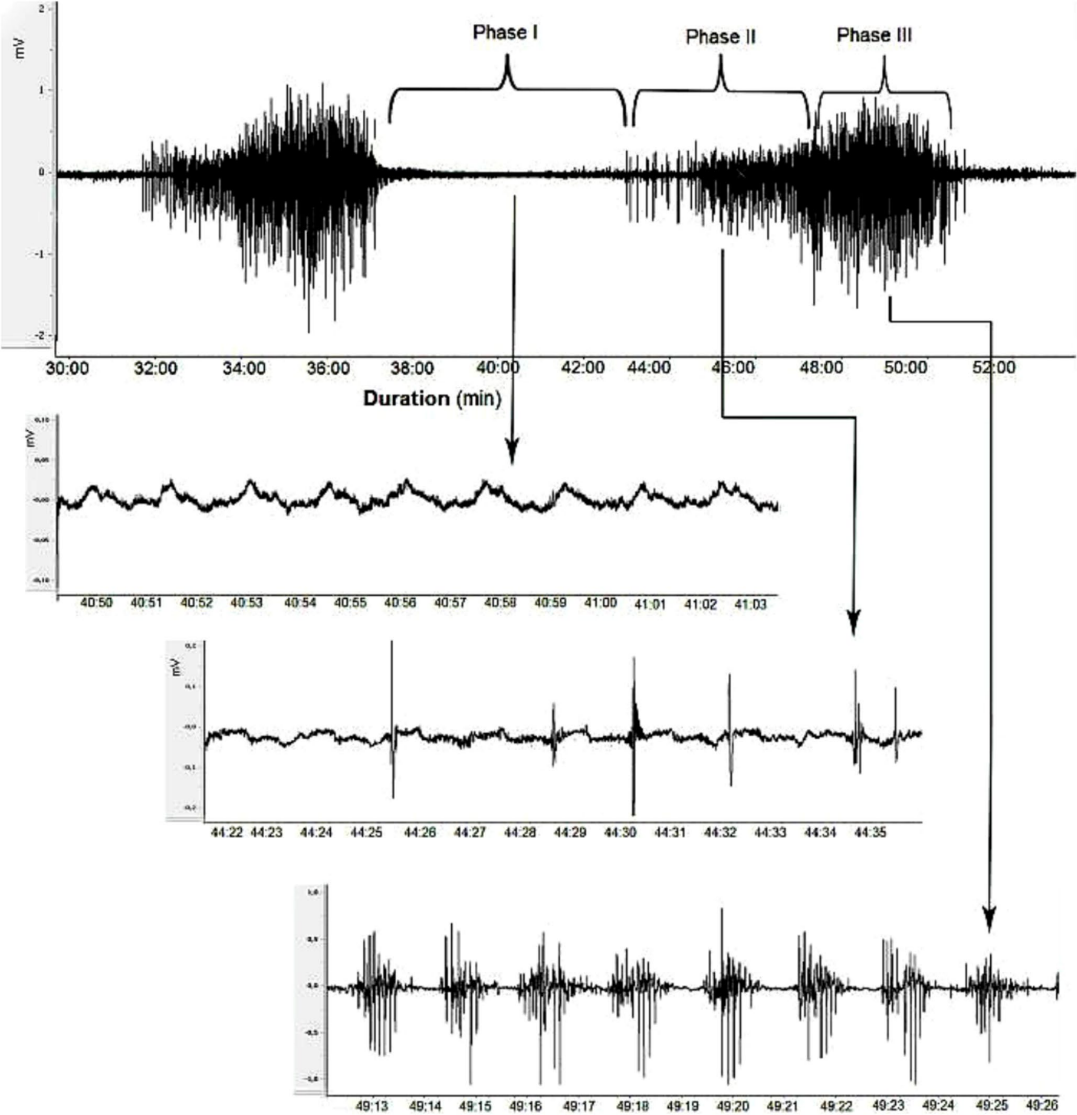
A representative myoelectric recording obtained in our laboratory, showing phases I, II, and III of MMC along with corresponding slow wave and spike wave activities

Typical intestinal myoelectric activity during the interdigestive period is characterized by a clearly distinguishable phase III, which propagates in the oral-to-anal direction along the intestinal segment, followed by phases I and II. Phase I, known as the silent period, is characterized almost exclusively by slow waves. Phase II is marked by the presence of spike waves superimposed on the peaks of certain slow waves. In contrast, phase III is defined by densely clustered spike wave activity on all slow waves. The transition from phase III to phase I continues a rhythmic, cyclic pattern that recurs consistently throughout the fasting state [15, 23, 44]. A representative recording from our dataset at the J1 site demonstrates the phases of the MMC—phases I, II, and III—along with slow wave and spike wave activities (Fig. 3). Spike counts and durations of MMC phases (phase I, II, III) and also MMC cycle at the J1 and J2 recording sites during the infusion period were calculated using LabChart 7.0 software. Additionally, the number of MMC cycles observed during this period was also determined. Spike potentials persisting longer than 30 min without regular cyclic activity were classified as disrupted MMC patterns or irregular spiking activity (ISA). In the analyses, the software defined electrical activities with amplitudes exceeding 0.15 mV as spikes (spike waves or action potentials) [7, 32, 46].

### Statistical analysis

All data are expressed as mean ± SEM with 7–9 data per group. Normality was assessed prior to analysis using the Shapiro-Wilk test. For in vitro experiments, data were analyzed using one-way repeated-measures ANOVA, as sequential drug applications were performed on the same intestinal preparations. Each intestinal preparation was considered as one experimental unit, and treatment condition was treated as a within-preparation factor. Multiple comparisons were performed using Tukey’s post hoc test. For in vivo experiments, data were analyzed using a mixed-effects model with restricted maximum likelihood (REML), where treatment was included as a fixed effect and subject as a random effect. Multiple comparisons were conducted using Dunnett’s test, and multiplicity-adjusted p-values were reported. Values of *P* < 0.05 were considered statistically significant. All statistical analyses and graphical representations were generated using GraphPad Prism version 8.0.2 (GraphPad Software, San Diego, CA, USA).

## Results

### In vitro experiments

#### Effect of SPX on intestinal contractility

**Fig. 4:**
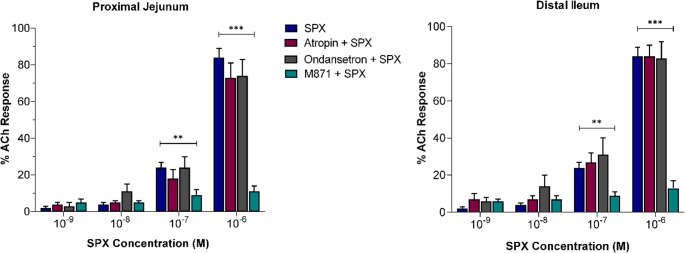
The left panel (proximal jejunum) and right panel (distal ileum) illustrate the effects of atropine, ondansetron, and M871 on SPX-induced contractile responses. Data are presented as percentages of the individual ACh-induced contraction and are expressed as group mean ± SEM. Significant differences compared to the corresponding SPX concentration are indicated with **p < 0.01 and ***p < 0.001

In the proximal jejunum, cumulative SPX administration at concentrations ranging from 10⁻⁹ M to 10⁻⁶ M induced contractions corresponding to 6 ± 2%, 6 ± 2%, 27 ± 5%, and 80 ± 7% of ACh-induced contraction, respectively. Similarly, in the distal ileum, SPX caused contractions equivalent to 2 ± 1%, 4 ± 1%, 24 ± 3%, and 84 ± 5% of ACh-induced contraction within the same concentration range (Fig. 4). At lower concentrations (10⁻⁹ M and 10⁻⁸ M), SPX did not induce notable contractile responses in either the proximal jejunum or distal ileum. In contrast, at higher concentrations (10⁻⁷ M and 10⁻⁶ M), SPX elicited evident contractile responses. These findings highlight the concentration-dependent nature of SPX-induced intestinal contractions.

#### The role of specific receptors in the SPX-induced intestinal contraction

Among the antagonists tested, M871 significantly suppressed SPX-induced contractions at 10⁻⁷ M and 10⁻⁶ M in both the proximal jejunum and distal ileum (Fig. 4).

In the proximal jejunum, responses were reduced to 9 ± 3% and 11 ± 3%, and in the distal ileum to 9 ± 2% and 13 ± 4%, respectively (*p* < 0.01–0.001). Conversely, atropine and ondansetron showed no statistically significant effects on SPX-induced contractions (Fig. 4).

### In vivo experiments

#### Effect of SPX on migrating myoelectric complex (MMC)

##### Durations of MMC phases and the total MMC cycle 

**Fig. 5:**
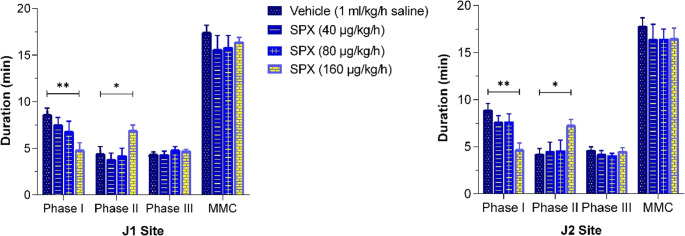
Effects of SPX (40, 80 and 160 µg/kg/h, IV infusion) on MMC phase (Phase I, II, III) durations, total MMC cycle duration at the J1 (left panel) and J2 (right panel) sites. The durations were analyzed under varying SPX doses and are expressed in minutes. All values are expressed as mean ± SEM (*n* = 7–9 per group). Significant differences compared to the vehicle group are indicated by *p < 0.05 and **p < 0.01

IV infusion of SPX at doses of 40 and 80 µg/kg/h did not result in statistically significant changes in the durations of MMC phases I, II, III, or the total MMC cycle in either intestinal region compared to the vehicle group. In contrast, the 160 µg/kg/h dose significantly shortened phase I duration at both the J1 (4.8 ± 0.8) and J2 (4.7 ± 0.7) sites compared to the vehicle group (J1: 8.6 ± 0.7, J2: 8.9 ± 0.7, *p* < 0.01). Conversely, this dose prolonged phase II at J1 (6.9 ± 0.6) and J2 (7.3 ± 0.6) relative to the vehicle group (J1: 4.4 ± 0.8, J2: 4.2 ± 0.6, *p* < 0.05). Notably, no significant changes were observed in phase III or the total MMC cycle durations (Fig. 5).

##### Spike counts 

**Fig. 6:**
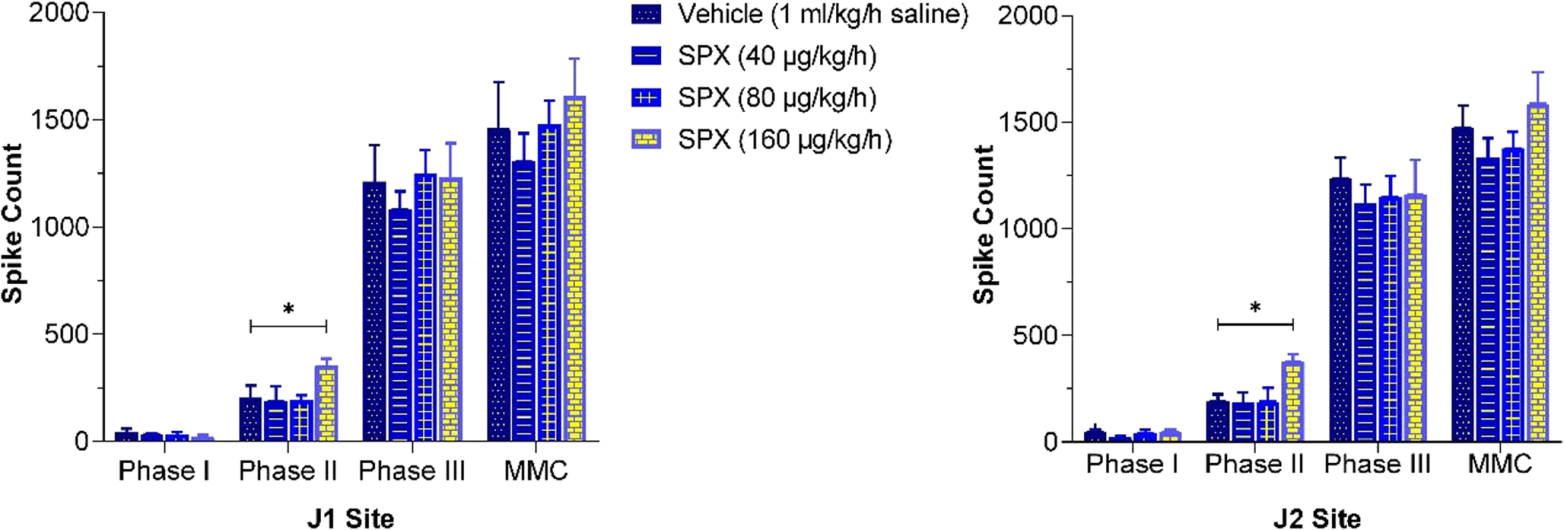
Effects of SPX (40–160 µg/kg/h, IV infusion) on spike counts during each MMC phase (Phase I, II, III) and total MMC cycle at the J1 (left panel) and J2 (right panel) intestinal recording sites. Spike counts were measured under varying SPX doses and represent the total number of spikes per phase or MMC cycle. Data are expressed as mean ± SEM (*n* = 7–9 per group). Significant differences compared to the vehicle group are indicated by *p < 0.05

SPX doses of 40 and 80 µg/kg/h did not significantly alter spike count during any phase at either intestinal site compared to the vehicle group. However, the 160 µg/kg/h dose significantly increased spike counts during phase II at J1 (358 ± 28) and J2 (376 ± 37) compared to the vehicle group (J1: 204 ± 57; J2: 190 ± 34, *p* < 0.05). No significant changes in spike counts were observed during other phases or throughout the MMC cycle at this dose (Figs. 6 and 8).

However, at higher SPX doses (320 and 640 µg/kg/h), the MMC pattern was replaced by ISA, which profoundly disrupted the normal cyclical rhythm. As a result, the durations and spike counts in each phase of the MMC could no longer be accurately determined at these SPX doses (Fig. 8; Table 1).

**Table 1:** Effect of receptor antagonists on SPX-induced MMC disruption

	Intestinal site	Duration (min)				Spike count				Number of MMC cycles (per hour)
		Phase I	Phase II	Phase III	MMC	Phase I	Phase II	Phase III	MMC	
*Vehicle (1 ml/kg/h saline)*	**J1**	8.6 ± 0.7	4.4 ± 0.8	4. 3 ± 0.3	17.4 ± 0.8	45 ± 15	204 ± 57	1212 ± 168	1460 ± 214	3.3 ± 0.1
	**J2**	8.9 ± 0.7	4.2 ± 0.6	4.6 ± 0.4	17.8 ± 0.9	48 ± 13	190 ± 34	1233 ± 99	1471 ± 107	3.3 ± 0.1
*SPX (320 µg/kg/h)*	**J1**	ISA				ISA				0
	**J2**	ISA				ISA				0
*SPX (640 µg/kg/h)*	**J1**	ISA				ISA				0
	**J2**	ISA				ISA				0
*Atropine (1 mg/kg)* *+* *SPX (320 µg/kg/h)*	**J1**	6.8 ± 0.2	5.1 ± 0.8	4.7 ± 0.5	16.6 ± 0.7	29 ± 7	282 ± 25	1223 ± 104	1578 ± 211	3.3 ± 0.3
	**J2**	7.1 ± 0.3	6.1 ± 1.2	4.8 ± 0.4	18 ± 0.8	31 ± 5	212 ± 33	1198 ± 124	1497 ± 205	3.2 ± 0.4
*M871 (1 mg/kg)* *+* *SPX (320 µg/kg/h)*	**J1**	7.9 ± 0.5	5.8 ± 1.6	4.3 ± 0.4	18 ± 2.5	13 ± 3	263 ± 112	1177 ± 155	1563 ± 218	3.2 ± 0.4
	**J2**	8.1 ± 1.2	5.1 ± 0.9	4.3 ± 0.7	17.5 ± 1.9	26 ± 10	292 ± 98	1251 ± 180	1669 ± 232	3.0 ± 0.3
*Ondansetron (0.5 mg/kg)* *+* *SPX (320 µg/kg/h)*	**J1**	ISA				ISA				0
	**J2**	ISA				ISA				0

Phase durations, spike counts, and the number of MMC cycles are expressed as mean ± SEM. Irregular Spiking Activity (ISA) refers to disorganized spike discharges that do not follow the typical MMC pattern, characterized by distinct Phase I, II, and III. Phase durations are reported in minutes, while spike counts represent the total spikes per phase or entire MMC cycle. MMC cycle numbers indicate the total number of cycles observed within one hour during SPX infusion

##### Number of MMC cycles 

**Fig. 7:**
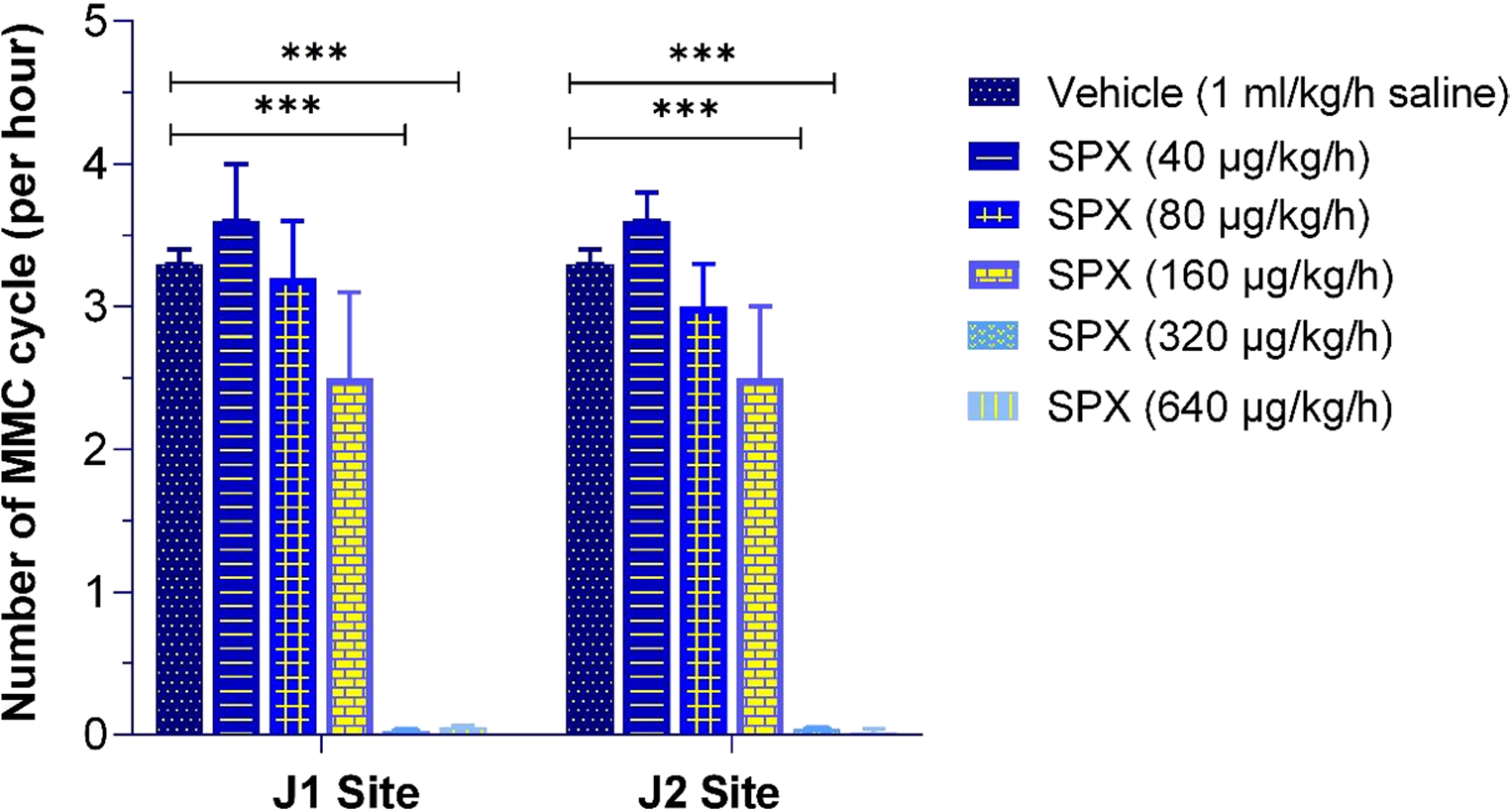
Effects of SPX (40, 80, 160, 320 and 640 µg/kg/h, IV infusion) on the number of MMC cycles at the J1 (left panel) and J2 (right panel) intestinal recording sites. The number of MMC cycles was analyzed across varying SPX doses and expressed as the number of cycles per hour. Data are presented as mean ± SEM (*n* = 7–9 per group). Significant differences compared to the vehicle group are indicated by ***p < 0.001

**Fig. 8:**
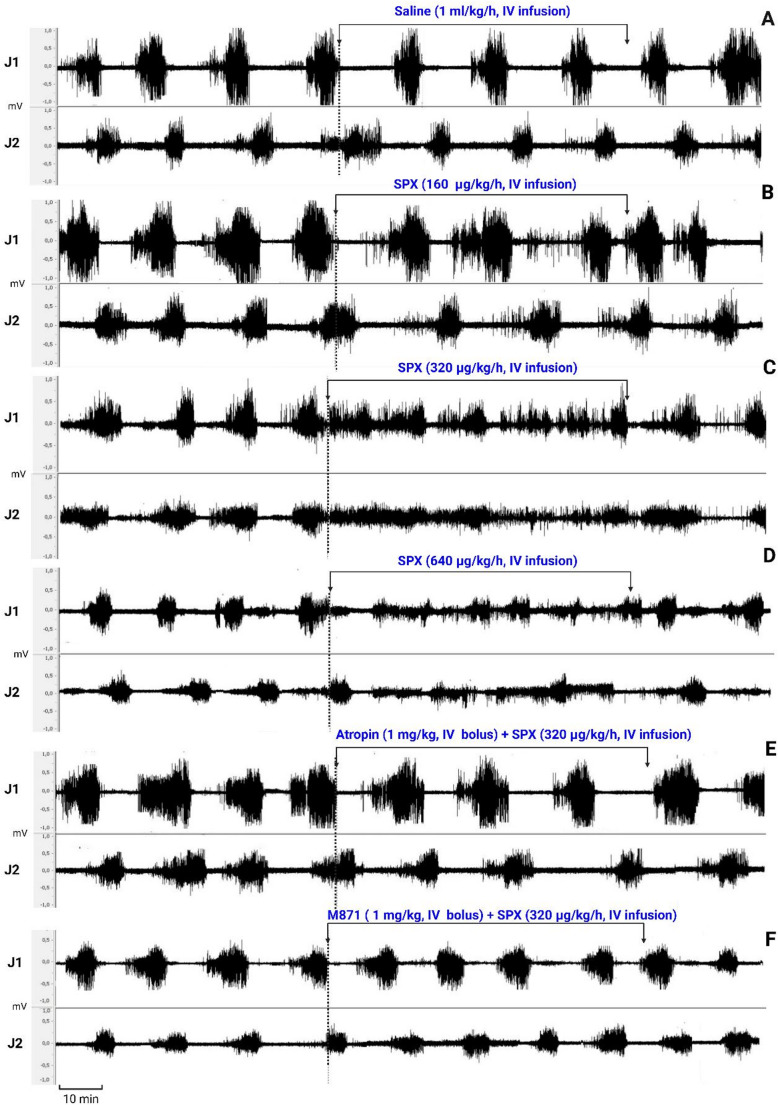
Representative recordings of intestinal myoelectric activity. The effects of intravenous infusion of vehicle (saline) at 1 mL/kg/h (Panel A, control condition), SPX at 160 µg/kg/h (Panel B), SPX at 320 µg/kg/h (Panel C), and SPX at 640 µg/kg/h (Panel D) are shown. Panels E and F demonstrate the effects of IV bolus pretreatment with atropine and M871, respectively, which completely prevented the SPX-induced changes at a dose of 320 µg/kg/h. The horizontal axis represents the timeline in minutes, while the vertical axis indicates the electrical activity amplitude (mV)

Consistent with the findings on durations and spike counts of MMC phases, SPX at doses of 40 and 80 µg/kg/h did not cause statistically significant changes in the number of MMC cycles compared to the vehicle group. The 160 µg/kg/h dose tended to reduce the number of MMC cycles; however, this effect did not reach statistical significance. In contrast, higher doses of SPX (320 and 640 µg/kg/h) completely disrupted normal MMC patterns, leading to the absence of detectable MMC cycles in all animals (Figs. 7 and 8; Table 1).

In summary, IV infusion of SPX exhibited dose-dependent effects on MMC patterns, with no significant impact observed at lower doses (40 and 80 µg/kg/h), partial alterations at 160 µg/kg/h, and complete disruption at higher doses (320 and 640 µg/kg/h), suggesting a potential role in modulating fasted intestinal motility. Moreover, the analysis revealed no statistically significant differences between the 320 and 640 µg/kg/h doses in terms of the number of MMC cycles, spike counts, or phase durations, indicating a plateau effect at higher doses (Figs. 7 and 8). Consequently, the 320 µg/kg/h dose of SPX was chosen for the combination groups.

### The role of specific receptors in SPX-induced modulation of MMC

The disruptive effect of SPX at 320 µg/kg/h on the MMC, characterized by the induction of ISA in fasted rats, was completely abolished by IV bolus atropine pretreatment (1 mg/kg). Similarly, the effect of SPX at this dose was fully blocked by pretreatment with M871 (1 mg/kg, IV bolus) (Fig. 8). As shown in Table 1, all evaluated parameters of MMC following these antagonist pretreatments were comparable to those observed in the vehicle group. In contrast, ondansetron pretreatment (0.5 mg/kg, IV bolus) failed to reverse the SPX-induced ISA in fasted rats (Table 1).

## Discussion

This study investigated the effects of SPX on small intestinal motility and its underlying regulatory mechanisms in both in vitro and in vivo conditions. In vitro experiments revealed that SPX induces concentration-dependent contractions in smooth muscle segments of the proximal jejunum and distal ileum, an effect mediated through GALR2s. In vivo analyses demonstrated that SPX alters MMC phase durations and spike activity, and at higher doses, disrupts the MMC cycle by inducing ISA, a disrupted motility pattern typically associated with postprandial states. In alignment with the in vitro findings, GALR2s mediated these in vivo effects. The selective involvement of muscarinic receptors in vivo, but not in vitro, is particularly noteworthy and suggests the recruitment of indirect regulatory pathways. However, no contribution of 5-HT_3_ receptors was observed in mediating the effects of SPX, either in vitro or in vivo. This study is the first to demonstrate the effects of SPX on fasting-phase intestinal motility and its underlying mechanisms involving GALR2 and muscarinic receptor pathways.

Our in vitro experiments demonstrated that SPX induces strong contractions in smooth muscle segments of the rat proximal jejunum and distal ileum at concentrations of 10⁻⁷ M and 10⁻⁶ M. These findings are consistent with the results reported by Mirabeau et al. (2007), who observed similar contractile responses in rat gastric tissue at comparable concentrations [34]. Similarly, Lin et al. (2015) showed that SPX produces dose-dependent contractions in mouse jejunal smooth muscle through activation of GALR2 [28]. Our findings confirm and extend previous studies by demonstrating that SPX induces contractile responses in both the jejunum and ileum through GALR2-mediated pathways, independently of muscarinic and serotonergic 5-HT_3_ receptors. Beyond these in vitro findings, our in vivo experiments demonstrated that SPX administration has substantial effects on the MMC. Administration of SPX at a dose of 160 µg/kg/h increased the number of spikes and prolonged the duration of phase II, while shortening phase I. At higher doses (320 and 640 µg/kg/h), SPX disrupted MMC cycling, inducing ISA, suggesting the induction of postprandial-like motility. Lin et al. (2015) reported that intraperitoneally administered SPX at similar high doses (300 and 1000 µg/kg) enhanced intestinal and colonic transit during the fed state in mice [28]. Extending these observations, our study demonstrated that the effects of SPX on MMC, particularly at 320 µg/kg/h, were completely abolished by receptor-specific antagonists such as atropine and M871. These findings suggest an important role for GALR2s and an indirect contribution of muscarinic pathways in mediating SPX-induced modulation of MMC activity.

GALR2s are broadly expressed throughout the gastrointestinal tract, with particularly high densities in the small intestine. These receptors are prominently localized within the muscularis externa and the myenteric plexus—structures critically involved in the regulation of intestinal motility [3, 4, 13, 27, 48]. GALR2 is known to mediate excitatory effects via G_q/11_-dependent signaling pathways, which facilitate smooth muscle contractions within the intestine [1, 3, 48]. This signaling mechanism is essential for the generation of rapid and robust contractile responses. Our results, consistent with the findings of Lin et al. (2015), demonstrated that GALR2-dependent mechanisms mediate the contractile activity induced by SPX in both isolated jejunum and ileum segments [28]. Given this information, we aimed to clarify the role of GALR2s in the effects of SPX on intestinal motility under in vivo conditions. In fasted rats, SPX administration disrupted MMC and promoted ISA through GALR2 activation. Taken together, our novel results indicate that GALR2s activation mediates not only the SPX-induced contractile effects in vitro but also its modulatory effects on the fasting intestinal motility (i.e., MMC). The GALR2s involved in the in vivo effects of SPX may be the same as those located in the intestinal wall that mediate SPX’s effects under in vitro conditions. However, GALR2s are also present outside the intestinal wall, and it is possible that these receptors may also contribute to the effects of intravenously administered SPX on the MMC. Unfortunately, our study is not sufficient to draw a definitive conclusion.

Muscarinic receptors are among the key regulatory components of the gastrointestinal system and play a crucial role in the control of intestinal motility. Indeed, muscarinic receptor involvement has been demonstrated in the disruption of the MMC and induction of ISA by various exogenously administered endogenous substances. For example, intravenous and intracerebroventricular injections of urocortin in fasted rats have been shown to disrupt small intestinal MMC and induce ISA via muscarinic pathways [53]. A similar muscarinic-dependent effect has been observed with centrally administered orexin, which induces both gastric and intestinal ISA in fasted rats [9]. Neuropeptide gamma has also been reported to disrupt MMC through muscarinic receptor-mediated mechanisms [38]. Consistent with these observations, in our in vivo experiments, the complete inhibition of SPX-induced disruptive effects on the MMC through pretreatment with the non-selective muscarinic receptor antagonist atropine indicates a significant contribution of muscarinic receptors to this response. Both intrinsic and extrinsic muscarinic receptors should be considered in relation to this effect. It is well established that intrinsic muscarinic receptors, located on enteric cholinergic motor neurons, interneurons, and smooth muscle, modulate contractile activity throughout the intestinal wall [19, 36, 42]. Our in vitro findings demonstrated that SPX-induced contractions were not altered by atropine, suggesting that intrinsic muscarinic receptors are not involved in this response. However, the same conclusion cannot be directly extended to the in vivo settings, since intravenously administered SPX may modulate additional extrinsic mechanisms, which could indirectly engage muscarinic receptors in the intestinal wall. In this context, brainstem structures such as the dorsal motor nucleus (DMN) and the nucleus tractus solitarius (NTS) may be relevant, as these regions contain muscarinic receptors and can modulate parasympathetic outflow to the intestine [8, 24, 25]. Accordingly, exogenously administered SPX may influence fasting intestinal activity via intrinsic and/or extrinsic muscarinic pathways. However, since our study did not include experiments specifically targeting central muscarinic receptors, the contribution of extrinsic pathways remains hypothetical.

The detection of GALR2 expression in the DMN [10], which mediates parasympathetic control of the gut [8], raises the possibility that SPX may potentially activate vagal efferent fibers by stimulating receptors in this region. Additionally, SPX may also interact with GALR2s located on the cell bodies of vagal afferent neurons within the nodose ganglion [54], which could, in turn, lead to the activation of vagal efferent neuron cell bodies within the DMN. This potential neural circuit bears similarity to previous findings showing that activation of vagal afferents via cholecystokinin-B receptors leads to disruption of the MMC and the emergence of ISA [39]. This pathway may also involve muscarinic receptors located in the brainstem—particularly in NTS and DMN—which have been shown to disrupt the MMC and induce ISA when stimulated [20]. However, it should be noted that these interpretations are based on indirect evidence and should be considered hypothetical. Collectively, these findings suggest that SPX may influence the enteric nervous system either directly or indirectly by activating the parasympathetic vagal efferent pathway, although the relative contribution of extrinsic neural mechanisms remains to be determined. Further experimental approaches, such as SPX administration following truncal vagotomy, would be required to clarify and validate these proposed mechanisms.

It is well established that 5-HT₃ receptors play a role in the regulation of MMC [15, 16, 40]. In light of these findings, blockade of 5-HT₃ receptors has been shown to prolong the MMC interval [5]. Lördal and Hellström (1999) reported that exogenous administration of serotonin increases the frequency of MMCs, whereas higher doses induce ISA [31]. Although their study demonstrated that the effect of serotonin on MMC frequency was mediated via 5-HT₃ receptors, the role of these receptors in serotonin-induced ISA has not been investigated. Indeed, to date, no studies have examined the involvement of 5-HT₃ receptors in the mechanism by which exogenous substances disrupt MMC and induce ISA. In this context, our study demonstrates that 5-HT₃ receptors are not involved in ISA induced by intravenously administered SPX. Further investigation into the contribution of other 5-HT receptor subtypes to SPX-induced changes in MMC may provide a more comprehensive understanding of the underlying mechanisms.

In conclusion, this study provides novel and systematic insights into the role of SPX in fasting intestinal motility. Our findings suggest that SPX modulates fasting intestinal motility through GALR2-dependent mechanisms, with an indirect contribution of muscarinic pathways. In these respects, our study is anticipated to encourage further research into the effects of SPX on intestinal motility and the mechanisms through which these effects are mediated. The results of these studies may facilitate the development of novel therapeutic approaches for treating motility-related gastrointestinal disorders.

## Data Availability

Data supporting the findings of this study are available from the corresponding author upon reasonable request.
